# Mindful understanding of the interconnected world: The synergistic effects of mindfulness and interconnectedness in driving collective action and autonomous motivation

**DOI:** 10.1111/bjop.70080

**Published:** 2026-05-08

**Authors:** Floria H. N. Chio, Ben C. L. Yu, Winnie W. S. Mak

**Affiliations:** ^1^ Department of Psychology The Chinese University of Hong Kong Shatin Hong Kong; ^2^ Department of Applied Social Sciences The Hong Kong Polytechnic University Hung Hom Hong Kong

**Keywords:** autonomous motivation, collective action, interconnectedness, mindfulness

## Abstract

The present study examined the roles of interconnectedness and mindfulness in collective action participation and the motivations underlying the participation. Two studies were conducted. In Study 1, 377 participants completed measures of mindfulness, interconnectedness, collective action intention and participation at baseline, with 308 and 279 participants completing follow‐up assessments 1 and 2 months later, respectively. Results showed that after accounting for baseline mindfulness, baseline interconnectedness has an indirect effect on collective action participation at 2‐month follow‐up through collective action intention at 1‐month follow‐up. Mindfulness showed no significant interaction effect with interconnectedness in predicting collective action intention or participation. In Study 2, 308 participants completed measures of mindfulness, interconnectedness, autonomous motivations and collective action at baseline, with 268 participants completing the 2‐month follow‐up assessment. Results showed that the effect of baseline interconnectedness on subsequent levels of autonomous motivations was contingent on baseline mindfulness, with mindfulness amplifying the beneficial effects of interconnectedness on autonomous motivations at 2‐month follow‐up. In addition, identified motivation was significantly associated with collective action at 2‐month follow‐up. These findings suggest that interconnectedness may serve as the primary intentional driver of collective action, whereas mindfulness may help translate interconnectedness into more autonomous motivation for participation.

## BACKGROUND

Socially minoritized communities, such as people with mental illness and sexual and gender diverse individuals, often face significant stress due to structural discrimination, systemic social exclusion and stigmatization (Meyer, 2003; Pescosolido et al., 2021; Thornicroft et al., 2009). These stressors and stigma can contribute to adverse psychological and social outcomes, including heightened psychological distress and reduced access to resources and life opportunities (Corrigan et al., 2009; Pellicane & Ciesla, 2022; Thornicroft, 2008). Given that these social injustices are entrenched societal problems prevailing across interpersonal and societal levels, mobilizing collective efforts is needed to effectively address these social issues. In addition to large‐scale collective actions, such as demonstrations, individuals can engage in private forms of collective action, involving everyday behaviours aimed at increasing awareness of social inequalities and fostering positive change within their immediate social circles. This can include challenging discriminatory remarks, addressing microaggressions or correcting stigmatizing language (Chan & Mak, 2021). Such private forms of collective action, which can be performed continuously in everyday life, play a vital role in challenging everyday injustices and laying the foundation for broader societal change.

### Mindfulness and its potential effect on collective action

Despite growing research on mindfulness and its benefits for well‐being, relatively few studies have explored its relationship with collective action but some studies have shown its positive association with prosociality (e.g., Donald et al., 2019). Mindfulness, characterized by the moment‐to‐moment awareness of the present moment with curiosity and openness (Bishop et al., 2004; Kabat‐Zinn, 1990), has been suggested to foster prosociality by enhancing sustained awareness, making individuals more attentive to others' needs (Donald et al., 2019). It also fosters emotion regulation, which helps reduce personal distress in response to others' suffering and allows for more compassionate and thoughtful helping behaviour (Donald et al., 2019).

Nevertheless, the effect of mindfulness on prosocial and justice‐oriented values is not consistent. Indeed, mindfulness may facilitate awareness and clarification of one's own values and help individuals behave in ways that are consistent with those values (Mak et al., 2021; Shapiro et al., 2006). Therefore, it is possible that mindfulness may not directly enhance collective actions, but its effects may rather interact with individuals' pre‐existing values. For instance, Cutler (2024) found that the impact of mindfulness in reducing racial prejudice and increasing support for equitable policies interacted with individuals' levels of social dominance orientation, which is individuals' preference for inequality among social groups (Pratto et al., 1994). Specifically, mindfulness promoted warmth towards racial minorities, reducing discriminatory intent and fostering support for equitable policies, but only among individuals low in social dominance orientation. In contrast, individuals high in social dominance orientation exhibited a reversed pattern, with mindfulness decreasing warmth, which further predicted more discriminatory intent and less support for equitable policies. Similarly, another study showed that mindfulness decreased prosociality among participants who were primed with independent self‐construal but increased prosociality among participants who were primed with interdependent self‐construal (Poulin et al., 2021). These findings suggest that while mindfulness may not directly enhance prosocial and justice‐oriented values, it amplifies individuals' pre‐existing values.

### Interconnectedness as an alternative predictor of collective action

Some studies suggest that additional elements need to be incorporated into the attention‐based practice of mindfulness to strengthen its effect on prosocial values (e.g. Yu et al., 2020). One such study examined how secular mindfulness, focussed on intentional awareness of the present moment without judgment, may differ from ethical mindfulness, which emphasizes the interdependence of all beings in addition to secular mindfulness (Chen & Jordan, 2020). The results indicated that participants were more likely to display prosocial behaviours in the ethical mindfulness condition compared to those in the secular or control conditions. Similarly, another study explored whether adding wisdom‐based practices, which focus on the insight of interdependence and the nature of existence, might have a stronger effect than mindfulness practices that only include mindfulness and compassion (Furnell et al., 2024). The findings revealed that participants who practiced all three components, mindfulness, compassion and interdependence showed greater improvements in social connectedness and prosocial tendencies than those practicing only mindfulness and compassion or those in the control group. These studies suggest that incorporating the insight of interdependence together with mindfulness may have a synergistic effect to promote prosocial behaviours.

Consistent with these previous studies on how the insights of interdependence may promote prosocial values (Chen & Jordan, 2020; Furnell et al., 2024), one cross‐sectional study showed that interconnectedness has incremental value over mindfulness in predicting social justice ideologies (Yu et al., 2020). Interconnectedness is a Buddhist‐derived construct defined as ‘an awareness that the existence of all phenomena in the world is the result of the fulfillment of different causes and conditions, in which no entity can substantiate independently without relying on other factors’ (Yu et al., 2020, p. 1239). Building on cross‐sectional research highlighting the link between interconnectedness and social justice ideologies (Yu et al., 2020), longitudinal studies and experimental study have also demonstrated that interconnectedness is associated with a stronger sense of civic duty, increased intention to participate and actual participation in collective action (Mak et al., 2022; Yu et al., 2021, 2024). Developing an awareness of interconnectedness may enhance individuals' recognition of their impact on broader societal issues and their role in driving change, including addressing challenges faced by minoritized groups. Supporting this perspective, research has found that when individuals adopt an interconnected viewpoint, or seeing themselves as contributing to the recovery process of people with mental illness, they tend to exhibit lower social distance towards this group over time (Yu et al., 2022, 2025). These findings suggest that interconnectedness may serve as a more consistent predictor of collective action compared to mindfulness, which appears to be more context‐dependent.

### Motivations behind collective action

While interconnectedness may foster a stronger sense of civic duty and participation in collective action, the sustainability of such engagement may depend on the underlying motivation driving these actions. According to the self‐determination theory (SDT), individuals vary in how they internalize the reasons for their actions, which in turn influences their persistence and well‐being (Deci & Ryan, 1985, 2008, 2012; Ryan, 2017). SDT differentiates between autonomous motivation, where actions are aligned with personal values or intrinsic interest, and controlled motivation, where actions are driven by external pressures or obligations. Autonomous motivation includes intrinsic motivation (acting for inherent satisfaction) and identified regulation (acting because the behaviour aligns with personally endorsed values). In contrast, introjected regulation involves internal pressures, such as guilt or the desire to maintain self‐worth. While typically considered less optimal and more controlled, some research has found that approach‐oriented introjected motivation (e.g. striving for social approval) is positively associated with engagement and positive affect and is considered more autonomous and less controlled than avoidance‐oriented introjected motivation (e.g. striving to avoid shame; Assor et al., 2009; Sheldon et al., 2017). Following Sheldon et al.'s (2017) framework, where approach‐oriented (positive) introjected motivation loads onto the autonomous motivation dimension alongside identified regulation and intrinsic motivation, the present study defines autonomous motivation to include all three forms: positive introjected (acting to maintain self‐approval), identified (value‐congruent action) and intrinsic (inherent satisfaction) motivation. This operationalization captures the relatively autonomous regulations predicting engagement persistence.

Autonomous motivation can be crucial for sustaining engagement in collective action. Individuals who act in alignment with their personal values tend to experience greater persistence, more positive affect and satisfaction (Howard et al., 2021). In contrast, those who act due to obligation or external pressure may be more prone to burnout and stress (Slemp et al., 2020). Research also shows that when intrinsic motivation is high, prosocial motivation enhances persistence and productivity, whereas when intrinsic motivation is low, prosocial motivation may be less effective or even counterproductive (Grant, 2008), highlighting the importance of fostering an internalized, self‐determined motivation for collective action. Given these distinctions and the importance of autonomous motivations, the present study aimed to examine how mindfulness and interconnectedness can contribute to these different forms of autonomous motivation for collective action, in addition to intention.

Although interconnectedness is associated with collective action, its ability to cultivate autonomous motivation for such action may depend on mindfulness. Mindfulness allows individuals to observe their thoughts, emotions and external circumstances with greater clarity and value clarification (Shapiro et al., 2006). By enhancing awareness, mindfulness may support recognition of how one's actions align with deeply held values, including the insights provided by interconnectedness. While interconnectedness may provide the insight by highlighting interdependence and thereby the societal impact of one's behaviour, mindfulness may serve as a facilitator that strengthens the translation of these insights into collective action. In addition, such awareness of the alignment between collective action and interconnectedness may foster autonomous motivation, as individuals recognize these actions as consistent with their own interconnectedness values. Therefore, consistent with previous studies showing that mindfulness amplifies the effects of pre‐existing values or orientations on prosocial behaviour rather than driving actions independently (Cutler, 2024; Poulin et al., 2021), we hypothesize that mindfulness would moderate the association between interconnectedness and collective action, including both intention and behaviour. Previous studies also show that interconnectedness predicts both collective action intention and behaviour (Mak et al., 2022; Yu et al., 2021, 2024). According to the Theory of Planned Behaviour, intention is the proximal predictor of behaviour, capturing individuals' plans and commitment to perform specific actions (Ajzen, 1991; Conner & Sparks, 2005). While intention predicts behaviour, autonomous motivation also shows a medium effect size in behaviour change (Sheeran et al., 2021) and contributes additional significant variance in some health behaviours even after accounting for intention (Hagger et al., 2014). Empirical work based on the SDT further indicates that autonomous motivation is associated with the maintenance of health behaviours (Vancampfort et al., 2015) and with persistence (Howard et al., 2021), highlighting its role in behavioural maintenance. Given intention's proximal role in behaviour adoption and autonomous motivation's importance for sustaining engagement, both pathways to behaviour are essential for understanding effective collective action.

### Aims of the study

While mindfulness enhances awareness and clarity of values, interconnectedness provides the insights that drive collective action. Together, both interconnectedness and mindfulness may interact to shape individuals' autonomous motivation to engage in collective action, influencing the intention, motivations and actual participation in such efforts. It is hypothesized that mindfulness amplifies (moderates) the association between interconnectedness and collective action to advocate for the rights of minoritized groups, as well as the intention and autonomous motivations behind such engagement. To test these hypotheses, two studies were conducted. Study 1 used a longitudinal design to examine how mindfulness and interconnectedness predict both intentions and actual participation in collective action among socially dominant groups for people with mental illness over 2 months. It is hypothesized that the mediation from interconnectedness to subsequent levels of collective action through intention would depend on the levels of mindfulness. Specifically, mindfulness is hypothesized to moderate the association between interconnectedness and subsequent levels of intention as well as between interconnectedness and subsequent levels of collective action. Given that actual behaviour of collective action was measured, collective action was examined in the context of specific targeted groups, including people with anxiety disorder and people with schizophrenia. Anxiety disorders and schizophrenia are examples of, respectively, common and severe mental illnesses, each subject to varying levels of societal stigma. Including both conditions in our study enables us to assess whether our findings are applicable across both prevalent and more serious forms of mental illness (Wood et al., 2014; Yu et al., 2021) and to examine if the hypothesized paths are consistent across contexts.

Study 2 focussed on the motivational aspect of collective action, examining the interaction effect of mindfulness by interconnectedness on autonomous motivations and collective action. An indirect effect from interconnectedness to collective action through autonomous motivation that depends on levels of mindfulness was also hypothesized. No specific main effect hypotheses were proposed for interconnectedness or mindfulness on autonomous motivation subtypes, given limited direct literature and our emphasis on their synergistic effects. Rather, we hypothesized that mindfulness would moderate the positive association between interconnectedness and all three forms of autonomous motivation (positive introjected, identified, intrinsic) as well as the association between interconnectedness and collective action, facilitating value‐aligned internalization of collective action. Collective actions for minoritized groups were examined in the second study to examine the hypothesized model towards the broader group of minoritized individuals in general. By examining both intention and autonomous motivation, the study sought to provide a more comprehensive understanding of how mindfulness and interconnectedness contribute to self‐determined collective actions across specific and general socially marginalized groups. The proposed models were presented in Figures 1 and 2.

**FIGURE 1:**
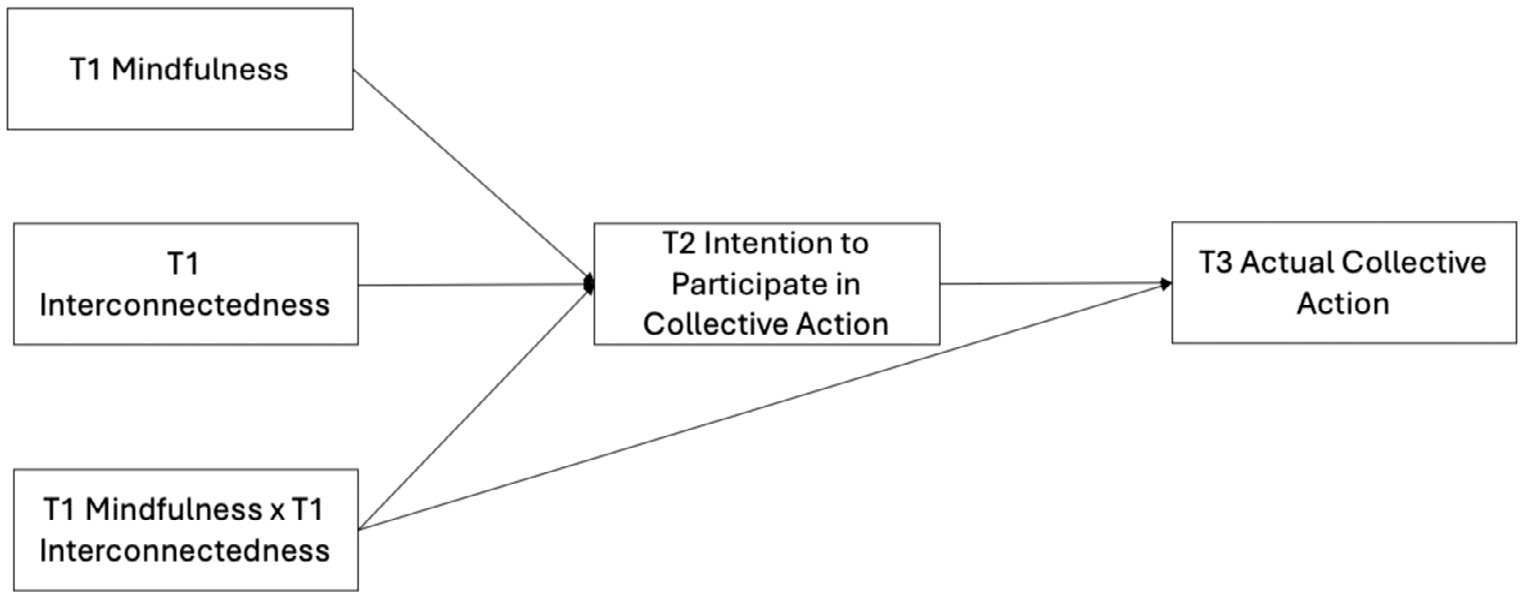
The proposed model in Study 1. T1 intention to participate in collective action, T1 actual collective action, prior mindfulness practice and prior contact with people with mental illness would be controlled.

**FIGURE 2:**
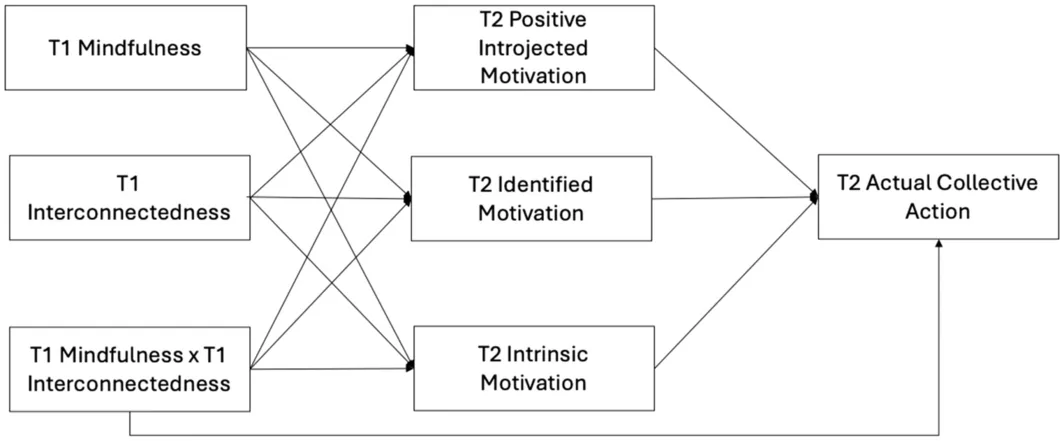
The proposed model in Study 2. T1 intention to participate in collective action, T1 actual collective action, prior mindfulness practice and prior contact with minoritized individuals would be controlled.

## STUDY 1

### Method

#### Participants

Data were collected from 377 students at a public university in Hong Kong (57% women; mean age = 21.55 years, *SD* = 5.15). Of these, 308 (82%) completed the 1‐month follow‐up, and 279 (74%) participated in the 2‐month follow‐up. Among those who remained at the final assessment, 57.3% were women. Most participants reported studying tertiary education or higher, with 81.4% being undergraduates and 16.8% pursuing postgraduate studies. Additionally, five participants (1.8%) reported having a diploma‐level education. A total of 15 participants (5.4%) indicated prior practice of mindfulness, and 112 of them (40.1%) indicated no contact with people with mental illness at the university, as neighbours, as close friends or as family. With Bonferroni adjustment in comparing differences, participants who were retained at the final assessment (*n* = 279) showed no significant differences from participants who dropped out (*n* = 98; *ts* <2.01, *ps* > .05; *χ* ^2^ < 10.64, *p*s > .22).

### Procedures

Data in the present study was from a larger study examining factors affecting stigma and collective action. Participants were recruited through mass email from a public university in Hong Kong. To be eligible, individuals had to be at least 18 years old, self‐reported having no history of clinically diagnosed mental illness and be able to read and understand written Chinese. After providing online informed consent, participants completed three online surveys over a 2‐month period using Qualtrics, an online survey platform. The surveys were administered at three time points: immediately after consent (T1), 1 month later (T2) and 2 months later (T3).

To enhance retention, email reminders were sent on Day 3 and Day 5 following the invitation for the 1‐month and 2‐month assessments. Participants who did not complete the surveys by Day 7 were considered missing for that time point. As compensation for the time spent, participants received HK$50 (HK$7.8 ≈ US$1) for each completed survey. Those who completed all three assessments received an additional HK$50, allowing a total of HK$200 (approximately US$25.6) for participation in the study. The study received approval from the Institutional Review Board of The Hong Kong Polytechnic before data collection began. The study was not pre‐registered.

### Measures

#### Mindfulness

The 15‐item Mindful Attention Awareness Scale (MAAS) (Brown & Ryan, 2003) was used to measure levels of mindfulness. A sample item is ‘I could be experiencing some emotion and not be conscious of it until some time later’. Each item was rated on a 6‐point Likert scale ranging from 1 (*Almost never*) to 6 (*Almost always*). The translated Chinese version of MAAS was validated with satisfactory internal reliability (Deng et al., 2012). The Cronbach's alpha of the scale was .88 in the present study.

#### Interconnectedness

The 12‐item Interconnectedness scale (Yu et al., 2020) was used to assess the participants' level of interconnectedness. A sample item is ‘I believe/think that the person I am today is a combination of past experiences’. Each item was rated on a 6‐point Likert scale from 1 (*totally disagree*) to 6 (*totally agree*). The scale was developed and validated in Hong Kong and has shown satisfactory reliability (Yu et al., 2020). The Cronbach's alpha of the scale in the present study was .78.

#### Intention to participate in collective action

The present study adapted the 3‐item Private Collective Action subscale from the Collective Action Scale (Chan & Mak, 2021) to assess individuals' intention to advocate for people with anxiety disorder or schizophrenia, respectively. For each time point, participants completed six items in total: three items referring explicitly to people with anxiety disorder and the same three items reworded to refer explicitly to people with schizophrenia. Participants rated their levels of willingness to engage in collective action on a 5‐point Likert scale (1 = not at all, 5 = very much). An example pair of items includes ‘Discussing mental health issues with family and/or friends to raise awareness of the rights of people with anxiety disorder’ and ‘Discussing mental health issues with family and/or friends to raise awareness of the rights of people with schizophrenia’. Item scores were averaged separately for each target group, such that higher scores indicated a stronger intention to support that specific group. The Cronbach's alpha of the scale ranged from .77 to .81, across baseline and 1‐month, follow‐up assessments for advocacy towards individuals with schizophrenia and anxiety disorder, respectively.

#### Actual collective action participation

To assess actual participation, the present study adapted the 3‐item Private Collective Action subscale from the Collective Action Scale (Chan & Mak, 2021; Yu et al., 2024). Participants completed two parallel 3‐item versions of this measure examining how frequently they engaged in collective actions supporting (a) people with anxiety disorder and (b) people with schizophrenia in the past month. Participants responded using a 5‐point Likert scale ranging from 1 (never) to 5 (frequently). One such action included ‘Discussing mental health issues with family and/or friends to raise awareness of the rights of people with anxiety disorder (or people with schizophrenia)’. Two separate composite scores were computed, one for each target group, with higher scores indicating greater engagement in private collective action over the past month. Reliabilities (Cronbach's alpha) ranged from .85 to .90 in the present study, across baseline and 1‐month, and 2‐month follow‐up assessments for advocacy towards individuals with schizophrenia disorder and anxiety disorder, respectively.

#### Prior mindfulness practice and contact with people with mental illness

Participants were asked if they have prior meditation experience. They were also asked to rate the amount of contact they had with people with mental illness at the university, as neighbours, as close friends and as family from (1) none at all to (7) a great deal. Sum scores from these four items were computed, with a higher score indicating higher levels of contact with people with mental illness.

### Data analysis

For the main analyses, the models were estimated using observed‐variable path analysis implemented in the PROCESS macro (Hayes, 2018) rather than latent‐variable structural equation modelling (SEM). Although SEM offers advantages such as explicitly modelling measurement error, path models require substantially smaller samples to achieve adequate power than their SEM counterparts (e.g., Sim et al., 2022). Assuming a medium effect‐size, a sample of approximately 110 is sufficient for a mediation model with two independent variables, one mediator and one outcome when estimated as a path model, whereas an SEM version of the same model would require a sample of roughly 430 with multiple indicators and moderate factor loadings. It has also been shown that for observed variables models, estimates obtained from PROCESS and from SEM software are largely similar, and SEM programmes may also be biased under smaller‐sample conditions (Hayes et al., 2017). Given these sample‐size considerations and the expectation of highly similar results between observed‐variable SEM programmes and PROCESS, PROCESS was selected as the tool for testing the hypothesized moderated mediation models. Missing data were handled with listwise deletion.

To assess the interaction effect of T1 interconnectedness and T1 mindfulness on subsequent levels of T2 collective action intention and T3 collective action, Model 8 was used to test the moderated mediation. Specifically, T1 interconnectedness was treated as the independent variable, T1 mindfulness as the moderator, T2 collective action intention as the mediator, and T3 collective action as the dependent variable. Prior contact with people with mental illness, prior meditation experience, T1 collective action intention and T1 collective action were included as covariates.

## RESULTS

### Differences in collective action intention and behaviour across target groups

Repeated‐measures analyses of variance were conducted to compare collective action intention and behaviour across the two target groups (people with anxiety disorder vs. people with schizophrenia). Significant differences were observed for collective action intention at T1 and T2 (T1: *F*(1, 278) = 36.91, *p* < .001, T2: *F*(1, 278) = 15.05, *p* < .001, T3: *F*(1, 278) = 0.65, *p* = .42), with higher intention observed when the target group is people with anxiety disorder. Similarly, collective action behaviour was significantly higher for people with anxiety disorder across times than people with schizophrenia (T1: *F*(1, 278) = 20.60, *p* < .001, T2: *F*(1, 278) = 17.02, *p* < .001, T3: *F*(1, 278) = 16.95, *p* < .001). These findings indicate that participants showed stronger intentions and behaviours when advocating for the less‐stigmatized group (people with anxiety disorder) relative to the more highly stigmatized group (people with schizophrenia).

### Interaction effect of interconnectedness and mindfulness on collective action intention and actual collective action behaviour

Results showed that the interaction effect of interconnectedness by mindfulness was non‐significant in predicting T2 collective action intention (*B* = 0.01, *SE* = 0.09, *p* = .95, 95% CI: [−0.17, 0.18]) and T3 actual collective action behaviour for people with anxiety disorder (*B* = −0.03, *SE* = 0.12, *p* = .80, 95% CI: [−0.26, 0.20]). Similarly, the interaction effect of interconnectedness by mindfulness was also non‐significant in predicting T2 collective action intention (*B* = 0.06, *SE* = 0.08, *p* = .42, 95% CI: [−0.09, 0.22]) and T3 collective action behaviour for people with schizophrenia (*B* = −0.15, *SE* = 0.12, *p* = .20, 95% CI: [−0.39, 0.08]). A summary of the parameter estimates of the interaction effect is shown in Table 1.

**TABLE 1:** Parameter estimates of the relative and interaction effects of mindfulness and interconnectedness on collective action intention and outcomes.

	Mediation model				Moderated mediation model			
	Collective action intention		Actual collective action		Collective action intention		Actual collective action	
	*B* (*SE*)	95% CI	*B* (*SE*)	95% CI	*B* (*SE*)	95% CI	*B* (*SE*)	95% CI
Interconnectedness	0.20 (0.08)*/0.16 (0.07)*	0.04, 0.35/0.02, 0.30	0.03 (0.11)/0.12 (0.11)	−0.24, 0.18/−0.09, 0.33	0.18 (0.26)/−0.02 (0.23)	−0.34, 0.70/−0.48, 0.44	0.05 (0.35)/0.55 (0.36)	−0.64, 0.75/−0.15, 1.25
Mindfulness	−0.08 (0.06)/−0.04 (0.05)	−0.19, 0.04/−0.14, 0.05	0.17 (0.08)*/0.24 (0.08)**	0.02, 0.32/0.09, 0.39	−0.10 (0.40)/−0.33 (0.35)	−0.88, 0.69/−1.02, 0.37	0.31 (0.53)/0.92 (0.54)	−0.74, 1.36/−0.14, 1.98
Collective action intention			0.35 (0.08)***/0.32, (0.09)***	0.19, 0.51/0.14, 0.50			0.35 (0.08)***/0.33 (0.09)***	0.19, 0.51/0.15, 0.51
Interconnectedness × Mindfulness					0.01 (0.09)/0.06 (0.08)	−0.17, 0.18/−0.09, 0.22	−0.03 (0.12)/−0.16 (0.12)	−0.26, 0.20/−0.39, 0.08

*Note*: Figure before and after the slash represents collective action intention and behaviour for people with anxiety disorder and schizophrenia, respectively. The baseline collective action intention, baseline actual collective action, prior mindfulness practice and prior contact with people with mental illness were controlled.

* *p* < .05;

** *p* < .01;

*** *p* < .001.

### Indirect effect of interconnectedness and mindfulness on actual collective action behaviour through collective action intention

The non‐significant moderation analyses suggested that the mediation may not depend on levels of mindfulness. Thus, mediation analyses were conducted to explore the mechanisms of interconnectedness and mindfulness on collective action through intention without the interaction term.

#### Collective action for people with anxiety disorder

Results showed that T1 mindfulness was not significantly associated with T2 collective action intention (*B* = −0.07, *SE* = 0.06, *p* = .19, 95% CI: [−0.19, 0.04]). However, a significant direct effect on collective action behaviour was found (*B* = 0.17, *SE* = 0.08, *p* = .03, 95% CI: [0.02, 0.32]). On the other hand, T1 interconnectedness was associated with T2 collective action intention (*B* = 0.20, *SE* = 0.08, *p* = .01, 95% CI: [0.04, 0.35]). T2 collective action intention was also found to be associated with T3 actual collective action (*B* = 0.35, *SE* = 0.08, *p* < .001, 95% CI: [0.19, 0.51]). The indirect effect from T1 interconnectedness to T3 collective action through T2 collective action intention was significant (*B* = 0.07, *SE* = 0.03, Boot CI: [0.02, 0.14]).

#### Collective action for people with schizophrenia

Results from mediation analysis showed that T1 mindfulness was not significantly associated with T2 collective action intention (*B* = −0.04, *SE* = 0.05, *p* = .38, 95% CI: [−0.14, 0.05]). While an indirect effect from mindfulness to collective action through intention was not observed, a significant direct effect was found (*B* = 0.24, *SE* = 0.08, *p* < .01, 95% CI: [0.09, 0.39]). On the other hand, T1 interconnectedness was significantly associated with T2 collective action intention (*B* = 0.16, *SE* = 0.07, *p* = .02, 95% CI: [0.02, 0.30]), which in turn was associated with T3 actual collective action (*B* = 0.32, *SE* = 0.09, *p* < .001, 95% CI: [−0.14, 0.50]). The indirect effect from T1 interconnectedness to T3 collective action through T2 collective action intention was also significant (*B* = 0.05, *SE* = 0.03, Boot CI: [0.004, 0.11]). A summary of the mediation effect of mindfulness and interconnectedness on collective action through collective action intention is shown in Table 1.

## STUDY 2

### Method

#### Participants

A total of 308 participants (70.1% women; mean age = 22.35 years, *SD* = 6.62 years) were recruited. A total of 268 (87%) valid responses were retained at 2‐month follow‐up (70.2% women; mean age = 22.41 years, *SD* = 6.93). A majority of the participants have had experiences or contact with social minoritized groups (63.4%) while most of them had never practiced mindfulness (72.0%). Participants who dropped out at 2‐month follow‐up (*n* = 40) did not show significant differences from participants who were retained (*n* = 268) on the baseline measures and demographic characteristics after Bonferroni adjustment (*t*s < 2.02, *p*s > .04; *χ* ^2^ < 2.66, *p*s > .10).

### Procedure

Ethics approval was obtained from The Chinese University of Hong Kong prior to the study commencement. Participants were recruited through the mass email system at a public university in Hong Kong. Inclusion criteria were as follows: (1) aged 18 or above, (2) owns an electronic device that has WhatsApp/Signal, which are two major online communication software used in Hong Kong, installed for reminder of follow‐up assessment purpose and (3) able to comprehend and understand Chinese. Participants who fulfilled all three criteria and had consented to participate in the study were invited to complete the online questionnaire and 2 months after the baseline questionnaire. Participants who completed both surveys were entered into a lucky draw for remuneration, with a total of 23 winners: three participants received HK$1000, five received HK$500, and 15 received HK$300 as compensation for their time and effort spent on the study. The study was not pre‐registered.

### Measures

#### Mindfulness and interconnectedness

The same mindfulness and interconnectedness scales as Study 1 were used. Cronbach's alpha of the mindfulness and interconnectedness scales was .87 and .80, respectively.

#### Actual collective action

The same 3‐item scale as Study 1 was used but the targeted group was changed to people with minoritized identities. Participants were asked to rate the items for minoritized identities and examples of minoritized groups were given, which included ethnicaly diverse individuals, sexually and gender diverse individuals, people with low income, people experiencing homelessness, and people with mental illness. Cronbach's alpha was .70 and .80 at baseline and 2‐month follow‐up, respectively.

#### Autonomous motivation

The Comprehensive Relative Autonomy Index (C‐RAI) (Sheldon et al., 2017) was adapted to measure one's motivational in advocating for social minoritized groups. Each item was rated on a 7‐point Likert scale from 1 (*Not true at all*) to 7 (*Very true*). The C‐RAI contains six subscales: amotivation, external motivation, negative introjection, positive introjection, identified motivation and intrinsic motivation. In the present study, the three subscales measuring autonomous motivation, namely positive introjection, identified motivation and intrinsic motivation, were used. A sample item is ‘because I strongly value advocating for minoritized groups’. C‐RAI has demonstrated satisfactory reliability across university samples (Sheldon et al., 2017). The Cronbach's alpha for positive introjected, identified and intrinsic motivation subscales at baseline and 2‐month follow‐up were .87, .82, .82, .89, .86 and .85, respectively.

#### Prior contact with minoritized groups and prior mindfulness practice

Participants were asked to indicate if they have prior contact with minoritized groups. They were also asked if they have prior mindfulness practice.

### Data analysis

To examine the hypothesized moderated mediation model, PROCESS (model 8; Hayes, 2018) was used. With 5000 bootstrapped samples, T1 interconnectedness, T1 mindfulness, T2 actual collective action and the three types of T2 motivations were treated as independent variables, the moderator, the dependent variable and the mediators, respectively. Prior contact with minoritized groups, prior mindfulness practice, T1 motivations and T1 actual collective action were treated as the covariates. Missing data were treated with listwise deletion.

## RESULTS

Table 2 shows a summary of the intercorrelations between variables. Interconnectedness showed a small correlation with identified and intrinsic motivation both at baseline and at 2‐month follow‐up, whereas mindfulness was not significantly correlated with any of the motivations.

**TABLE 2:** Intercorrelations between variables in Study 2.

	Mean (*SD*)	1	2	3	4	5	6	7	8	9
1. T1 Interconnectedness	4.50 (0.61)	1.00								
2. T1 Mindfulness	4.06 (0.80)	0.23***	1.00							
3. T1 Introjected positive motivation	2.58 (0.97)	0.04	−0.07	1.00						
4. T1 Identified motivation	3.37 (0.82)	0.14*	0.06	0.32***	1.00					
5. T1 Intrinsic motivation	3.22 (0.84)	0.14*	0.02	0.49***	0.59***	1.00				
6 T1 Actual collective action	2.96 (0.88)	0.12	−0.03	0.17**	0.47***	0.33***	1.00			
7. T2 Introjected positive motivation	2.51 (0.92)	0.04	−0.08	0.54***	0.18**	0.29***	0.16^**^	1.00		
8. T2 Identified motivation	3.23 (0.84)	0.17**	0.08	0.29***	0.57^*^ **	0.44***	0.39***	0.37***	1.00	
9. T2 Intrinsic motivation	3.05 (0.87)	0.15*	0.02	0.40^*^ **	0.47**	0.55***	0.27***	0.58***	0.66***	1.00
10. T2 Actual collective action	2.86 (0.95)	0.10	−0.09	0.21^*^ **	0.43***	0.35***	0.63***	0.30***	0.48***	0.40***

* *p* < .05;

** *p* < .01;

*** *p* < .001.

Results from PROCESS showed that the interaction term of interconnectedness and mindfulness was significant in predicting all three types of motivations. In particular, when mindfulness was 1 *SD* above the mean, significant positive associations between interconnectedness and identified motivation (*B* = 0.32, *SE* = 0.10, *p* = .001, 95% CI [0.13, 0.52]) as well as between interconnectedness and intrinsic motivation (*B* = 0.33, *SE* = 0.10, *p* = .001, 95% CI: [0.13, 0.53]) were found. The association between interconnectedness and positive introjected motivation approached significance when mindfulness was high (*B* = 0.21, *SE* = 0.11, *p* = .06, 95% CI: [−0.01, 0.44]). The associations between interconnectedness and the three types of motivations were non‐significant when mindfulness was low. T2 positive introjected motivation (*B* = 0.13, *SE* = 0.06, *p* = 0.04, 95% CI: [0.003, 0.26]) and identified motivation (*B* = 0.23, *SE* = 0.07, *p* = .002, 95% CI: [0.08, 0.38]) further predicted T2 collective action. However, intrinsic motivation was non‐significant in predicting T2 collective action (*B* = 0.06, *SE* = 0.08, *p* = .45, 95% CI: [−0.10, 0.22]). The interaction effect of interconnectedness and mindfulness on T2 collective action was non‐significant (*B* = −0.04, *SE* = 0.07, *p* = .57, 95% CI [−0.19, 0.10]). The index of moderated mediation is only significant on identified motivation (Index = 0.05, Boot *SE* = 0.03, Boot CI: [0.01, 0.11]), with the mediation from interconnectedness to collective action through identified motivation being significant when mindfulness was high (*B* = 0.07, Boot *SE* = 0.04, Boot CI: [0.02, 0.16]). Details of the parameter estimates are shown in Table 3.

**TABLE 3:** Parameter estimates of the moderator analysis in Study 2.

Variables	T2 Positive introjected motivation		T2 Identified motivation		T2 Intrinsic motivation		T2 Actual collective action	
	*B* (*SE*)	95% CI	*B* (*SE*)	95% CI	*B* (*SE*)	95% CI	*B* (*SE*)	95% CI
T1 interconnectedness	0.07 (0.08)	−0.09, 0.23	0.15 (0.07)*	0.01, 0.29	0.14 (0.07)	−0.001, 0.29	0.003 (0.07)	−0.14, 0.15
T1 mindfulness	−0.05 (0.06)	−0.17, 0.07	0.04 (0.05)	−0.07, 0.14	0.009 (0.05)	−0.12, 0.10	−0.10 (0.06)	−0.21, 0.01
T2 positive introjected motivation							0.13 (0.06)*	0.003, 0.26
T2 Identified motivation							0.23 (0.07)**	0.08, 0.38
T2 Intrinsic motivation							0.06 (0.08)	−0.10, 0.22
T1 Interconnectedness × T1 mindfulness	0.18 (0.08)*	0.02, 0.34	0.22 (0.07)**	0.08, 0.36	0.23 (0.07)**	0.09, 0.37	−0.04 (0.07)	−0.19, 0.10
Conditional effect								
Low mindfulness	−0.08 (0.09)	−0.26, 0.11	−0.03 (0.08)	−0.19, 0.13	−0.04 (0.08)	−0.21, 0.12		
Moderate mindfulness	0.07 (0.08)	−0.09, 0.23	0.15 (0.07)*	0.01, 0.29	0.14 (0.07)	−0.001, 0.29	.	
High mindfulness	0.21 (0.11)	−0.01, 0.44	0.32 (0.10)**	0.13, 0.52	0.33 (0.10)**	0.13, 0.53		

*Note*: the baseline motivations, baseline actual collective action, prior mindfulness practice and prior contact with minoritized individuals were controlled; low, moderate and high mindfulness was defined as 1 *SD* below mean, at mean level and 1 *SD* above mean, respectively.

* *p* < .05;

** *p* < .01.

## DISCUSSION

The present study examined the roles of interconnectedness and mindfulness in predicting collective action and their effects on autonomous motivation. Findings suggested that interconnectedness may be the primary intentional driver of collective action, relative to mindfulness. Importantly, this study extended previous research by demonstrating, using a prospective design, that interconnectedness remained a significant predictor of actual participation in collective action through collective action intention, after accounting for mindfulness. By fostering an awareness of the interdependent nature of individuals and their surroundings, interconnectedness enables people to recognize their responsibility and role in addressing societal injustices (Yu et al., 2025), which may drive their intention to act. Given previous findings on its association with collective action engagement and advocacy (Mak et al., 2022; Yu et al., 2021, 2024), interconnectedness may be a direct determinant of people's behavioural intention to participate in collective action.

Contrary to our hypotheses that mindfulness would primarily moderate rather than directly predict collective action, T1 mindfulness showed significant direct effects on T3 behaviour in Study 1, even after controlling for intention and interconnectedness, yet showed no association with intention itself. This suggests that mindfulness may promote collective action through mechanisms beyond deliberate planning. For instance, mindfulness may enhance present‐moment awareness and clarify personal values (Shapiro et al., 2006), enabling individuals to act in alignment with those values spontaneously without necessarily engaging in prior, explicit intention. In addition, Study 1 revealed that interconnectedness predicted collective action intention and subsequent behaviour consistently across both target groups (anxiety disorder and schizophrenia), despite significant differences in baseline intention and engagement in collective actions. The indirect pathway from interconnectedness to collective action through intention remained significant regardless of target, and mindfulness did not moderate this pathway. This finding suggests that the insight of interconnectedness may facilitate intention formation and collective action in a way that generalizes across these social groups who encountered varying levels of stigma.

While previous research has reported mixed findings on the link between mindfulness and prosocial behaviour, often moderated by traits or pre‐existing values (e.g., Cutler, 2024; Poulin et al., 2021), Study 2 reveals how mindfulness moderates the motivational pathway between interconnectedness and collective action for minoritized groups. Specifically, the association between interconnectedness and actual behaviour was significantly mediated by autonomous motivation, particularly identified motivation, and this indirect effect was present at higher levels of mindfulness. This moderation effect indicates that translating interconnectedness into self‐determined motivation for action may require mindfulness as a facilitating mechanism. Mindfulness appears to enhance individuals' ability to recognize how interconnected values align with their personal identities, thereby fostering more autonomous (self‐determined) motivation rather than externally driven obligation.

Taken together, the findings suggest a complementary yet distinct role of interconnectedness and mindfulness in collective action. Interconnectedness may provide the core insight that supports intention formation and subsequent collective action across groups with varying levels of stigma, as demonstrated in Study 1 with people with anxiety disorder and schizophrenia. Mindfulness may further amplify the effect of interconnectedness by facilitating the translation of this insight into action that is guided by more autonomous motivation. While Study 1 demonstrated that the intention‐based mediation pathway from interconnectedness to collective action operated similarly across two specific target groups, Study 2 examined a motivational pathway that assumed a more generalized context and focused on minoritized groups broadly. Given this difference in scope, future research is needed to examine whether the motivational processes identified in Study 2 vary across specific minoritized groups and to what extent mindfulness differentially shapes these pathways across contexts.

Our results further showed that while mindfulness moderated the effect of interconnectedness on all three types of motivation, only identified motivation predicted actual collective action. This is consistent with prior research suggesting that identified motivation, rather than intrinsic motivation, more reliably predicts behaviours that require discipline and sustained effort, such as voting or organizational citizenship (Gagné & Deci, 2005; Koestner et al., 1996; Van den Broeck et al., 2021). Collective action is rarely undertaken for enjoyment, as would be expected if driven by intrinsic motivation. Rather, it often demands effort and discipline. In this context, identified motivation may be particularly critical, as it reflects actions driven by personally endorsed values, even when the behaviour itself is effortful. Taken together, interconnectedness may explain who intend to engage in collective action, while mindfulness may shape why they engage by facilitating value clarification, resulting in a more autonomous motivation behind the participation.

These findings have implications for ways of mobilizing collective action and social change. While fostering a sense of interconnectedness alone may be sufficient to motivate deliberate intention to participate, integrating mindfulness practices may offer additional, longer‐term benefits. Given the protective role of autonomous motivation against burnout and disengagement (Hardre & Reeve, 2003; Slemp et al., 2020), combining mindfulness and interconnectedness could possibly help sustain engagement, particularly among social activists or individuals regularly involved in collective action. Mindfulness‐based training could explicitly cultivate awareness of interdependence, using guided exercises that prompt reflection on one's role in collective well‐being. By deepening individuals' connection to their values and the broader social context, this synergy may foster a more self‐determined and enduring commitment to social causes.

Nevertheless, this study has several limitations. First, the reliance on self‐reported data may introduce biases, such as social desirability effects or inaccuracies in how participants assess their motivations and behaviours. Second, the study sample consisted primarily of college students, which may limit the generalizability of the findings. Future research should examine whether these relationships hold across diverse populations, including individuals from different socio‐economic backgrounds or among people engaging in social activism. Third, the use of listwise deletion to handle missing data was used in the PROCESS models, which did not fully utilize all available information from participants with partial follow‐up data. Although attrition was non‐systematic with respect to baseline demographics and key variables, and bootstrapping enhanced inference robustness, future studies employing SEM with full information maximum likelihood estimation and larger samples could provide complementary robustness checks. Fourth, the two studies differed in their timing of assessments. Study 1 collected data across three waves (baseline, 1‐month follow‐up and 2‐month follow‐up), allowing intention to be measured at an intermediate time point before the outcome behaviour. Study 2, in contrast, used a two‐wave design (baseline and 2‐month follow‐up), with both autonomous motivation and collective action behaviour measured at the same follow‐up. Although this design still allowed us to examine the motivational pathway, it limits temporal interpretation of the mediation effect in Study 2. A further limitation is Study 2's broad ‘social minoritized groups’. While examples of minoritized groups were given in the instruction, it is possible that participants may have different interpretations based on their lived experience or social location. Future research may examine specific minoritized groups. Finally, while the study employed a longitudinal design, causality cannot be inferred. Experimental studies that manipulate mindfulness and interconnectedness interventions could provide stronger evidence for how these factors interact to influence collective action participation and motivation.

Overall, this study highlighted the crucial role of interconnectedness in driving collective action and the moderating role of mindfulness in contributing to the promotion of autonomous motivation. While interconnectedness predicts participation through intention, mindfulness enhances the self‐determined nature of individuals' engagement by reinforcing their alignment with collective action. The motivation that arises from an awareness of interconnectedness, bolstered by mindfulness, may serve as a powerful force in sustaining long‐term engagement in social justice efforts to advocate for the rights and welfare of minoritized groups in the society. These findings offer new insights for research and practice, emphasizing the need to integrate mindfulness and interconnectedness in efforts to promote collective action and foster positive systemic change for social sustainability.

## AUTHOR CONTRIBUTIONS

**Floria H. N. Chio:** Conceptualization; investigation; writing – review and editing; formal analysis; writing – original draft. **Ben C. L. Yu:** Funding acquisition; conceptualization; writing – review and editing. **Winnie W. S. Mak:** Conceptualization; supervision of data collection; funding acquisition; writing – review and editing.

## CONFLICT OF INTEREST STATEMENT

The authors declare no conflict of interest.

## COMPLIANCE WITH ETHICAL STANDARDS

The study described in this manuscript was approved by the appropriate human ethics committee and was performed in accordance with the ethical standards laid down in the Declaration of Helsinki.

## INFORMED CONSENT

Informed consent was obtained from all participants.

## Data Availability

The data that support the findings of this study are available on request from the corresponding author. The data are not publicly available due to privacy or ethical restrictions.
